# Supplementary material for: Comprehensive molecular characterization of pediatric radiation-induced high-grade glioma
Source: Nat Commun. 2021 Sep 20;12:5531. doi: 10.1038/s41467-021-25709-x (PMC8452624; doi:10.1038/s41467-021-25709-x)
Supplement: Supplementary file 1 — Supplementary Information [file 41467_2021_25709_MOESM1_ESM.pdf]

Comprehensive molecular characterization of pediatric radiation-induced high-grade glioma

Supplementary Information

DeSisto *et al.*

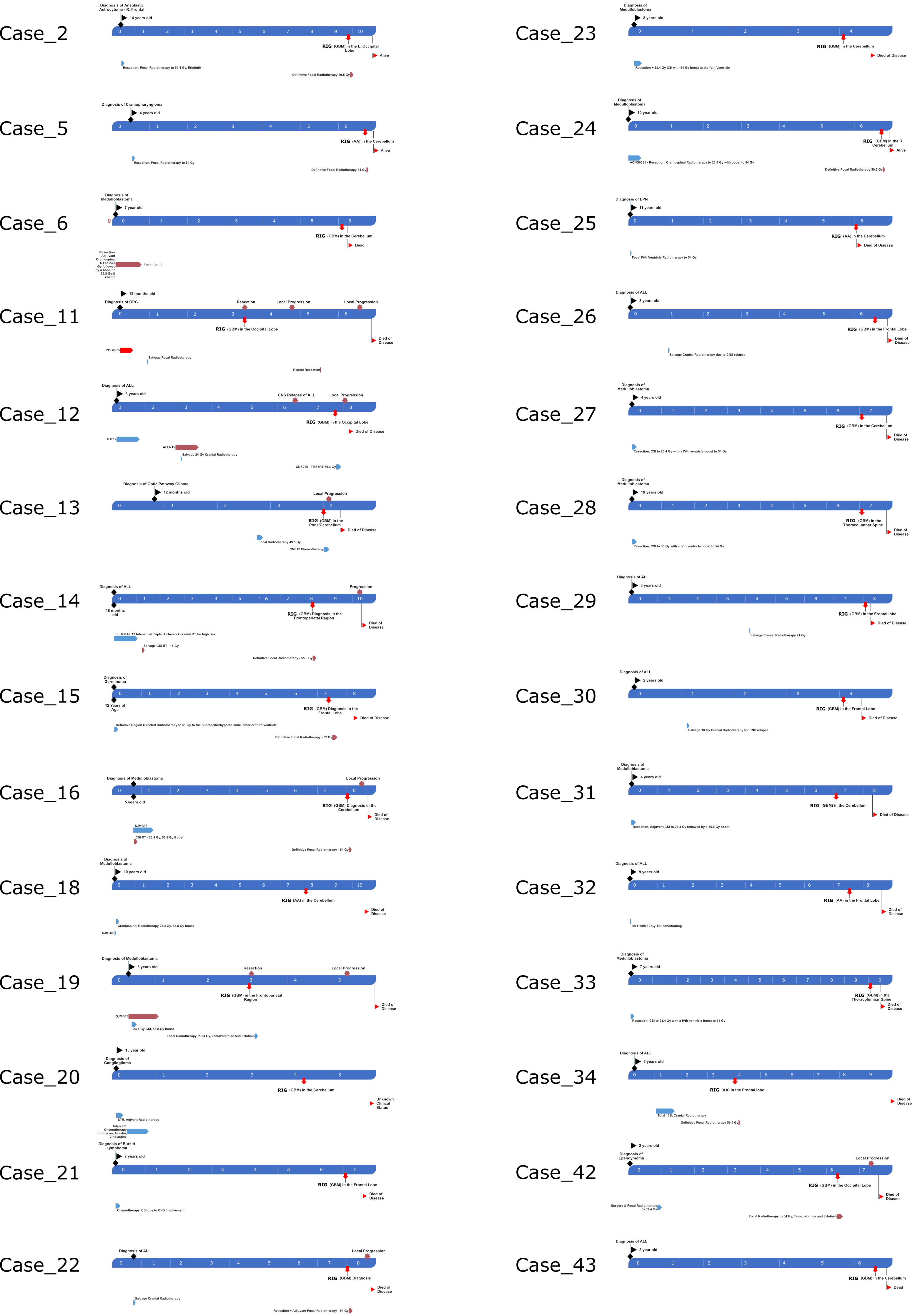

Case_2
Diagnosis of Anaplastic Astrocytoma - R. Frontal
14 years old
RIG (GBM) in the L. Occipital Lobe
Alive
Resection, Focal Radiotherapy to 59.4 Gy, Erlotinib
Definitive Focal Radiotherapy 59.5 Gy
Case_5
Diagnosis of Craniopharyngioma
4 years old
RIG (AA) in the Cerebellum
Alive
Resection, Focal Radiotherapy to 54 Gy
Definitive Focal Radiotherapy 54 Gy
Case_6
Diagnosis of Medulloblastoma
7 year old
RIG (GBM) in the Cerebellum
Dead
Resection, Adjuvant Craniospinal RT to 23.4 Gy followed by a boost to 55.8 Gy & chemo
Feb 4 - Oct 12
Case_11
12 months old
Diagnosis of OPG
Resection
Local Progression
Local Progression
RIG (GBM) in the Occipital Lobe
Died of Disease
POG8935
Salvage Focal Radiotherapy
Repeat Resection
Case_12
Diagnosis of ALL
3 years old
CNS Relapse of ALL
Local Progression
RIG (GBM) in the Occipital Lobe
Died of Disease
TOT12
ALLR15
Salvage 24 Gy Cranial Radiotherapy
CNS226 - TMZ+RT 59.4 Gy
Case_13
Diagnosis of Optic Pathway Glioma
12 months old
Local Progression
RIG (GBM) in the Pons/Cerebellum
Died of Disease
Focal Radiotherapy 49.5 Gy
CNS12 Chemotherapy
Case_14
Diagnosis of ALL
Progression
18 months old
RIG (GBM) Diagnosis in the Frontoparietal Region
Died of Disease
SJ TOTAL 13 Intensified Triple IT chemo + cranial RT for high risk
Salvage CSI RT - 18 Gy
Definitive Focal Radiotherapy - 55.8 Gy
Case_15
Diagnosis of Germinoma
12 Years of Age
RIG (GBM) Diagnosis in the Frontal Lobe
Died of Disease
Definitive Region Directed Radiotherapy to 51 Gy to the Suprasellar/hypothalamic, anterior third ventricle
Definitive Focal Radiotherapy - 52 Gy
Case_16
Diagnosis of Medulloblastoma
Local Progression
5 years old
RIG (GBM) Diagnosis in the Cerebellum
Died of Disease
SJMB96
CSI RT - 23.4 Gy, 55.8 Gy Boost
Definitive Focal Radiotherapy - 54 Gy
Case_18
Diagnosis of Medulloblastoma
10 years old
RIG (AA) in the Cerebellum
Died of Disease
Craniospinal Radiotherapy 23.4 Gy, 55.8 Gy boost
SJMB03
Case_19
Diagnosis of Medulloblastoma
8 years old
Resection
Local Progression
RIG (GBM) in the Frontoparietal Region
Died of Disease
SJMB03
23.4 Gy CSI, 55.8 Gy boost
Focal Radiotherapy to 54 Gy, Temozolomide and Erlotinib
Case_20
15 year old
Diagnosis of Ganglioglioma
RIG (GBM) in the Cerebellum
Unknown Clinical Status
STR, Adjuvant Radiotherapy
Adjuvant Chemotherapy Irinotecan, Avastin, Vinblastine
Case_21
Diagnosis of Burkitt Lymphoma
7 years old
RIG (GBM) in the Frontal Lobe
Died of Disease
Chemotherapy, CSI due to CNS involvement
Case_22
Diagnosis of ALL
Local Progression
RIG (GBM) Diagnosis
Died of Disease
Salvage Cranial Radiotherapy
Resection + Adjuvant Focal Radiotherapy - 54 Gy
Case_23
Diagnosis of Medulloblastoma
8 years old
RIG (GBM) in the Cerebellum
Died of Disease
Resection + 23.4 Gy CSI with 54 Gy boost to the IVth Ventricle
Case_24
Diagnosis of Medulloblastoma
10 year old
RIG (GBM) in the R. Cerebellum
Alive
ACNS0331 - Resection, Craniospinal Radiotherapy to 23.4 Gy with boost to 54 Gy
Definitive Focal Radiotherapy 59.5 Gy
Case_25
Diagnosis of EPN
11 years old
RIG (AA) in the Cerebellum
Died of Disease
Focal IVth Ventricle Radiotherapy to 54 Gy
Case_26
Diagnosis of ALL
3 years old
RIG (GBM) in the Frontal Lobe
Died of Disease
Salvage Cranial Radiotherapy due to CNS relapse
Case_27
Diagnosis of Medulloblastoma
4 years old
RIG (GBM) in the Cerebellum
Died of Disease
Resection, CSI to 23.4 Gy with a IVth ventricle boost to 54 Gy
Case_28
Diagnosis of Medulloblastoma
19 years old
RIG (GBM) in the Thoracolumbar Spine
Died of Disease
Resection, CSI to 36 Gy with a IVth ventricle boost to 54 Gy
Case_29
Diagnosis of ALL
3 years old
RIG (GBM) in the Frontal lobe
Died of Disease
Salvage Cranial Radiotherapy 21 Gy
Case_30
Diagnosis of ALL
2 years old
RIG (GBM) in the Frontal Lobe
Died of Disease
Salvage 18 Gy Cranial Radiotherapy for CNS relapse
Case_31
Diagnosis of Medulloblastoma
4 years old
RIG (GBM) in the Cerebellum
Died of Disease
Resection, Adjuvant CSI to 23.4 Gy followed by a 55.8 Gy boost
Case_32
Diagnosis of ALL
9 years old
RIG (AA) in the Frontal Lobe
Died of Disease
BMT with 12 Gy TBI conditioning
Case_33
Diagnosis of Medulloblastoma
7 years old
RIG (GBM) in the Thoracolumbar Spine
Died of Disease
Resection, CSI to 23.4 Gy with a IVth ventricle boost to 54 Gy
Case_34
Diagnosis of ALL
6 years old
RIG (AA) in the Frontal lobe
Died of Disease
Total 13B, Cranial Radiotherapy
Definitive Focal Radiotherapy 59.4 Gy
Case_42
2 years old
Diagnosis of Ependymoma
Local Progression
RIG (GBM) in the Occipital Lobe
Died of Disease
Surgery & Focal Radiotherapy to 59.4 Gy
Focal Radiotherapy to 54 Gy, Temozolomide and Erlotinib
Case_43
Diagnosis of ALL
2 year old
RIG (GBM) in the Cerebellum
Dead

Supplementary Figure 1. RIG case treatment histories. Antecedent cancer diagnosis is indicated as a black diamond. Age at diagnosis of the antecedent cancer is indicated adjacent to a black flag. Therapy for the antecedent cancer and RIG are indicated as horizontal teal and red bars, respectively, with description adjacent to each bar. RIG cancer diagnosis and histology are indicated as downward red arrows. Resection events are shown as burgundy diamonds. Progression events are shown as burgundy stars. RIG, radiation-induced high-grade glioma.

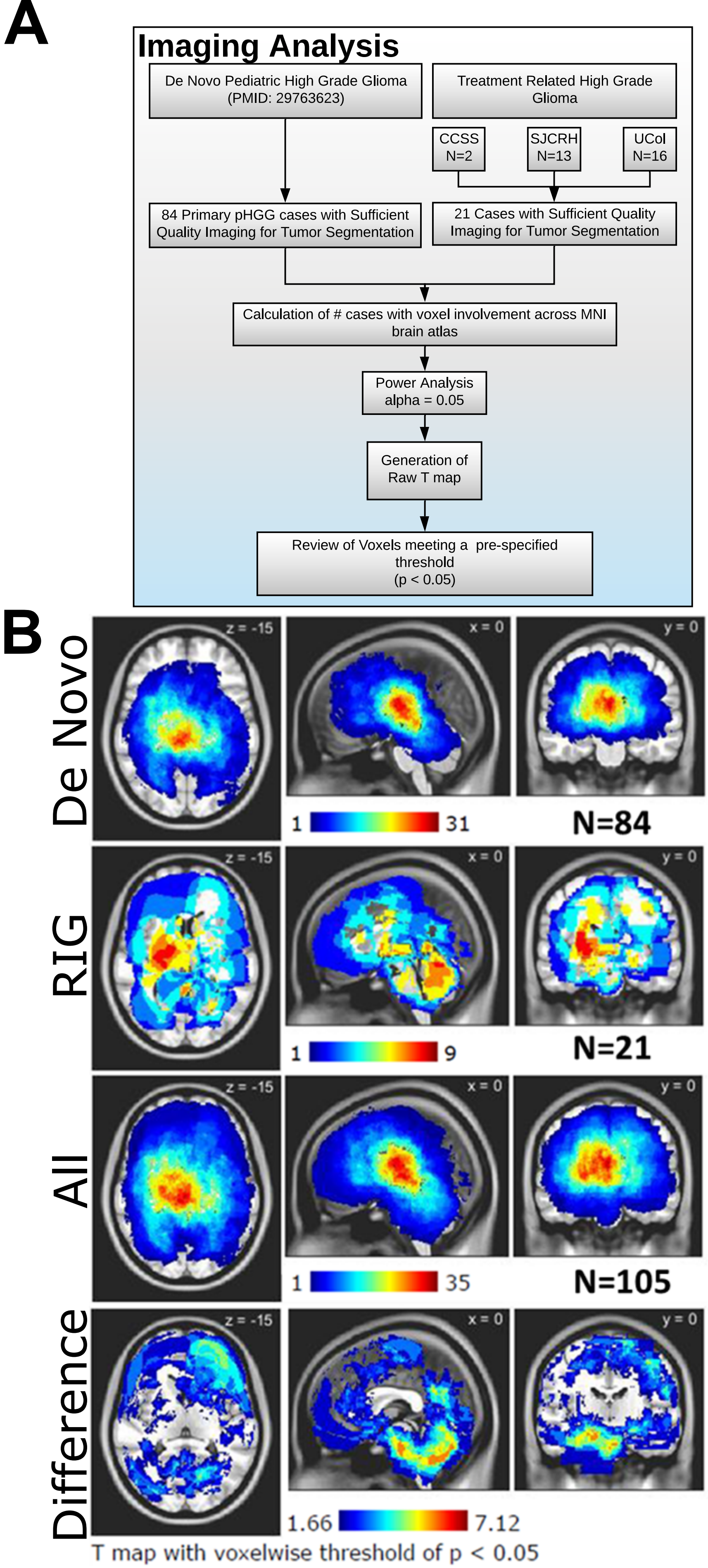
A
Imaging Analysis
De Novo Pediatric High Grade Glioma (PMID: 29763623)
Treatment Related High Grade Glioma
CCSS N=2
SJCRH N=13
UCol N=16
84 Primary pHGG cases with Sufficient Quality Imaging for Tumor Segmentation
21 Cases with Sufficient Quality Imaging for Tumor Segmentation
Calculation of # cases with voxel involvement across MNI brain atlas
Power Analysis alpha = 0.05
Generation of Raw T map
Review of Voxels meeting a pre-specified threshold (p < 0.05)
B
De Novo
z = -15
x = 0
y = 0
1
31
N=84
RIG
1
9
N=21
All
1
35
N=105
Difference
1.66
7.12
T map with voxelwise threshold of p < 0.05

Supplementary Figure 2. Spatial localization of RIG and *de novo* pHGG from the HERBY trial. A) Consort diagram illustrating the spatial analysis conducted to identify the locations in MNI brain space of the RIG and *de novo* HERBY cases at initial presentation. B) (Top Row) Distribution of 88 *de novo* HERBY pHGGs; (2nd Row) Distribution of 18 RIGs; (Row 3) Combined distribution of both *de novo* pHGG and RIGs; (Bottom Row) The spatial differences in distribution between *de novo* pHGGs and RIG using VLSM are shown with the corresponding frequency of difference events shown colorimetrically ($p<0.05$). The p-value threshold is calculated using a two-sample, two-sided t-test and is corrected for multiple comparisons. MNI, Montreal Neurological Institute; RIG, radiation-induced high-grade glioma; pHGG, pediatric high-grade glioma; VLSM, voxel-based lesion mapping. RIG, radiation-induced high-grade glioma.

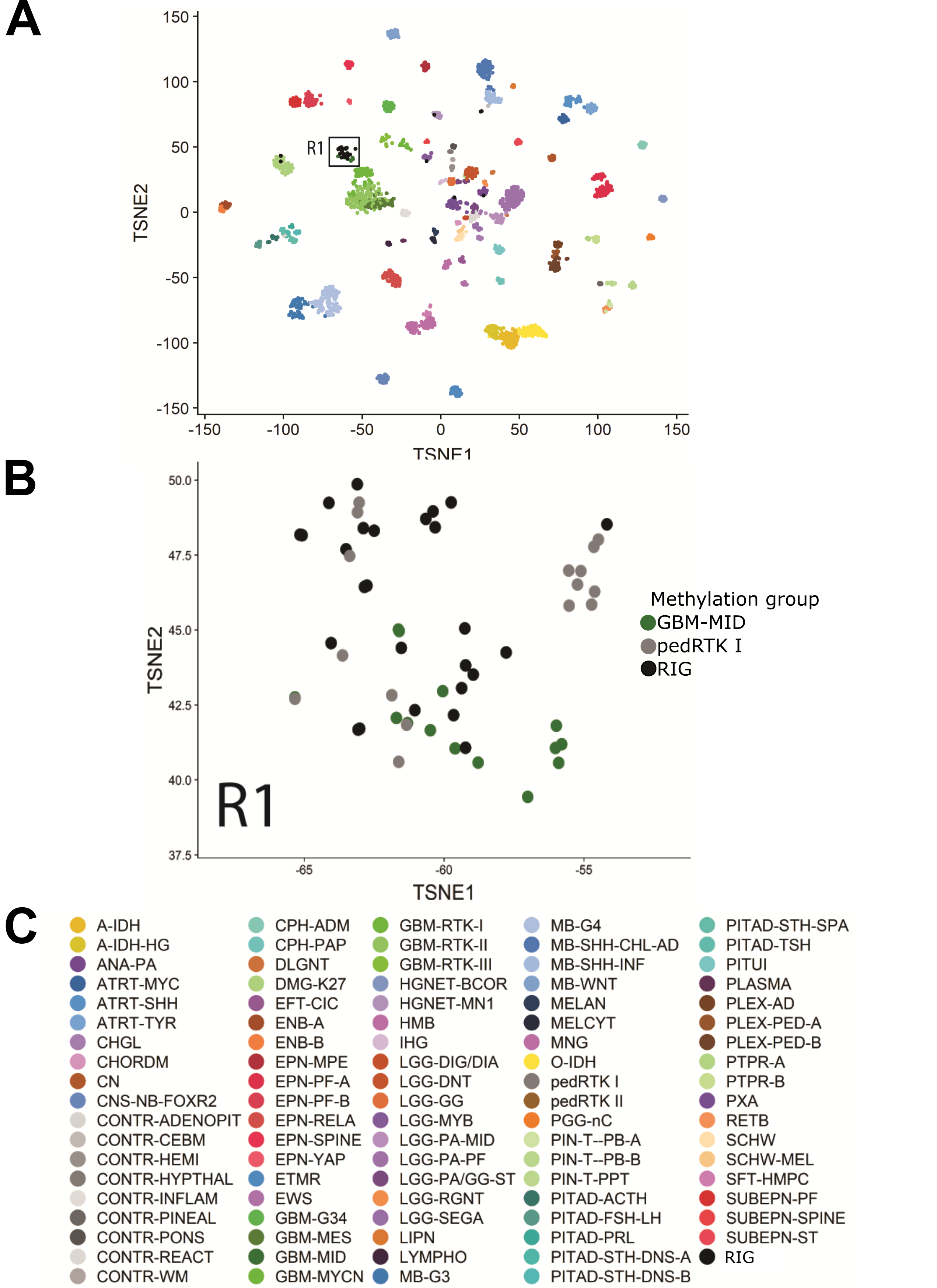

Supplementary Figure 3. Position of RIG among *de novo* CNS tumors based on methylation data. A) Localization of RIG tumors (R1) relative to other CNS cancers in t-SNE space. B) Magnified region indicated by R1 illustrating the close clustering relationship among GBM-MID, PedRTK I, and RIG tumors. C) Diagnostic methylation group key. CNS, central nervous system; RIG, radiation-induced high-grade gliomas.

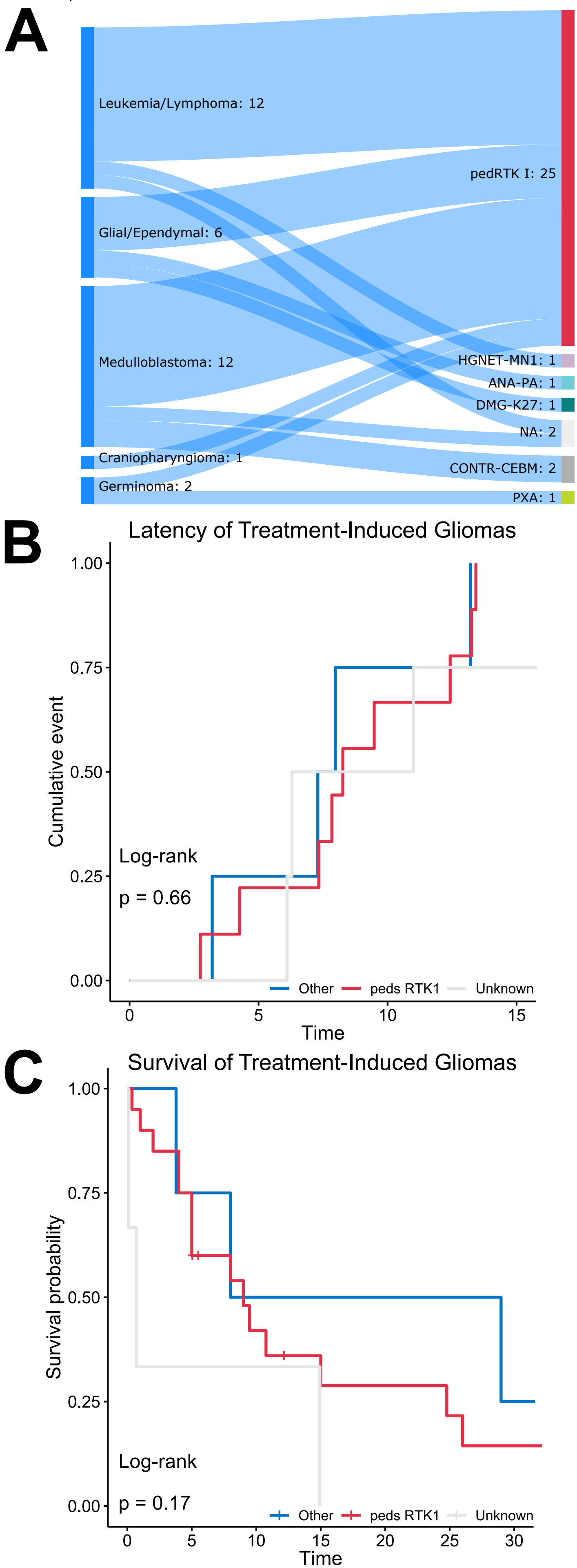

Supplementary Figure 4. Latency, outcomes, and relationship of antecedent cancer to RIG methylation class A) Sankey diagram illustrating the relationship of the antecedent cancer (left side) to the RIG methylation group (right side). B) Latency of RIG from the date of diagnosis of the antecedent cancer (in years) stratified by methylation group (pedRTK1, other, or unknown) (log-rank test). C) Overall survival probability of RIG cases stratified by pedRTK I vs. other vs. unknown (in months) from the date of RIG diagnosis. No significant trends were observed for the inter-relationship of antecedent cancer diagnosis, latency, or RIG patient survival and methylation subgroup (log-rank test). RIG, radiation-induced high-grade gliomas. All *p*-values are for two-sided tests.

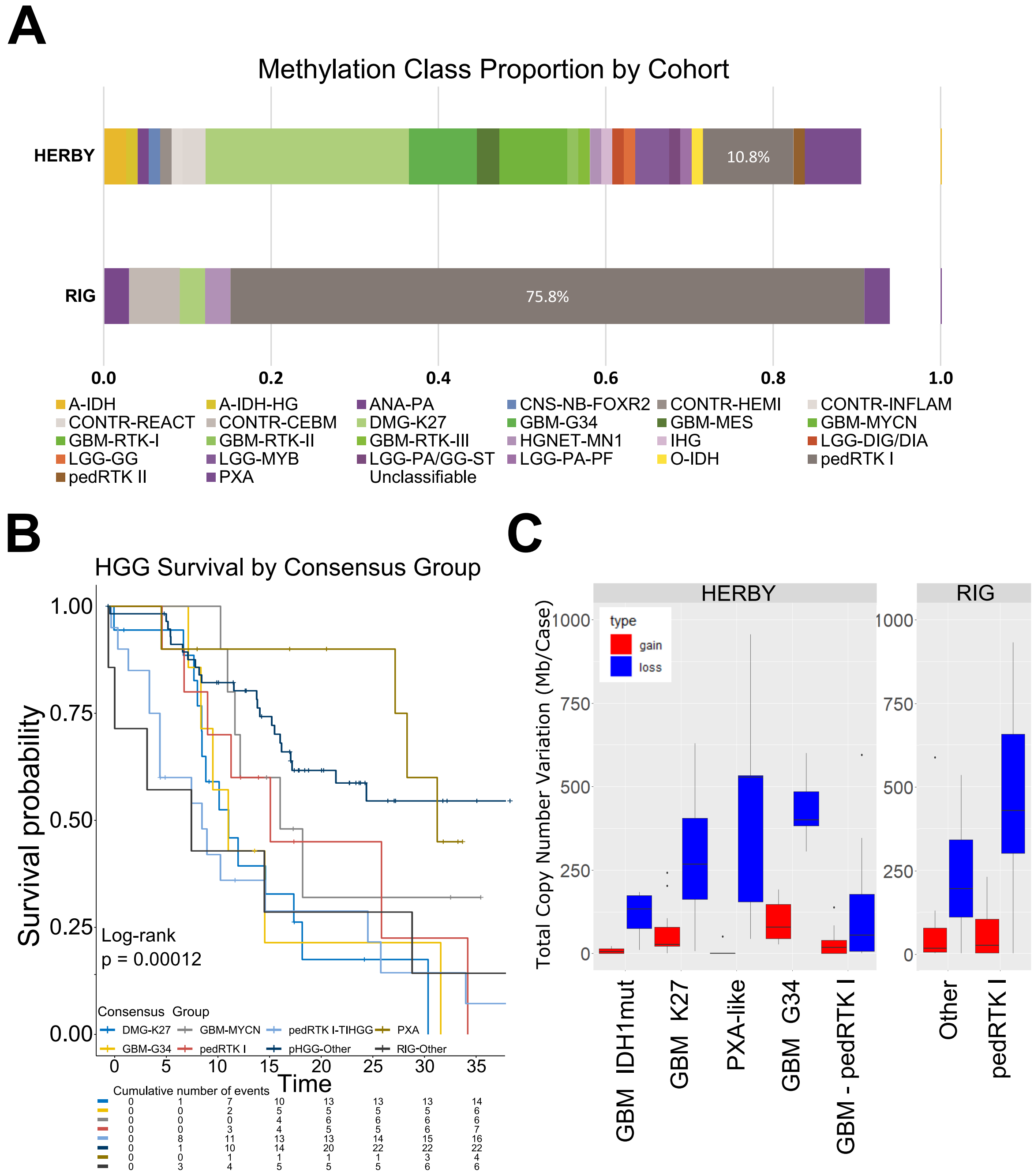

Supplementary Figure 5. Comparison of RIG to *de novo* pHGG from the HERBY cohort based on consensus methylation subgroup. A) Proportion of consensus cluster subgroups of RIG and *de novo* pHGG cases from the HERBY dataset. The pedRTK I subgroup predominates in the RIG cohort (75.8%) but constitutes a much smaller fraction (10.8%) of the HERBY cohort, in which the DMG-K27 and GBM RTK-I and II subgroups predominate. B) Survival of RIG and pHGG stratified by consensus methylation group (in months). RIG cases have comparable survival estimates relative to other poor performing *de novo* pHGG subgroups (DMG-K27, GBM-G34). Two-sided log-rank test. C) Total genomic length (Mb) lost and gained by methylation subgroup stratified by *de novo* pHGG (HERBY) and RIG; boxes show median and first and third quartiles, with whiskers representing range limited to 1.5× the interquartile range from the box edge. RIG, radiation-induced high-grade gliomas; pHGG, pediatric high-grade gliomas; GBM, glioblastoma. In panel c the median is represented by the central bar while the 1st and 3rd quartile are represented by the flanking bounds of the box, the range of the values is indicated by the bounds of the perpendicular line for each box and the solitary points represent outliers.

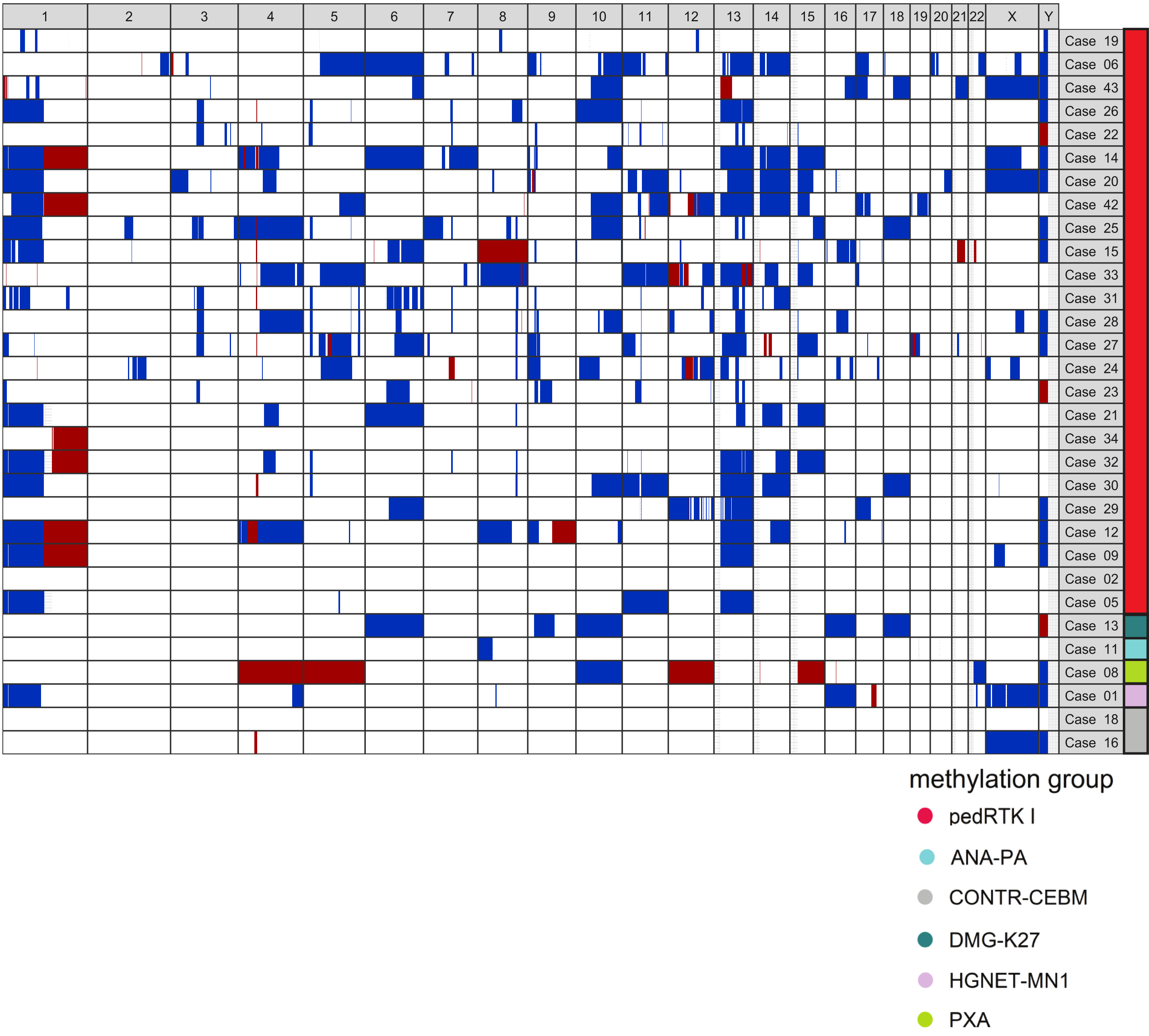

Supplementary Figure 6. Spectral plot depicting chromosome-level copy-number alterations (horizontal axis) for RIG samples (vertical axis). Consensus methylation groups are indicated on the vertical column adjacent to the case ID. Blue bars indicate copy-number loss, and red bars indicate copy-number gain. RIG, radiation-induced high-grade gliomas.

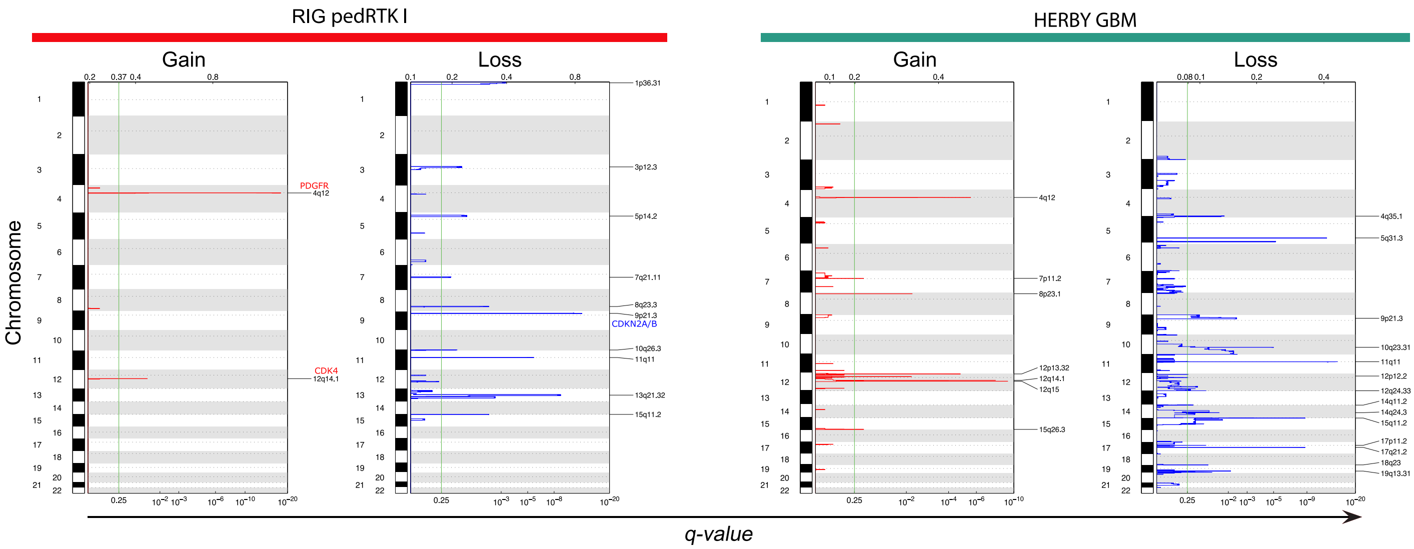

Supplementary Figure 7. Copy number gains and deletions in pedRTK I (a) and HERBY II GBM cohorts (b). Amplifications are shown in red while deletions are shown in blue. Green line indicates the *q*-value threshold (0.25) to be considered significant.

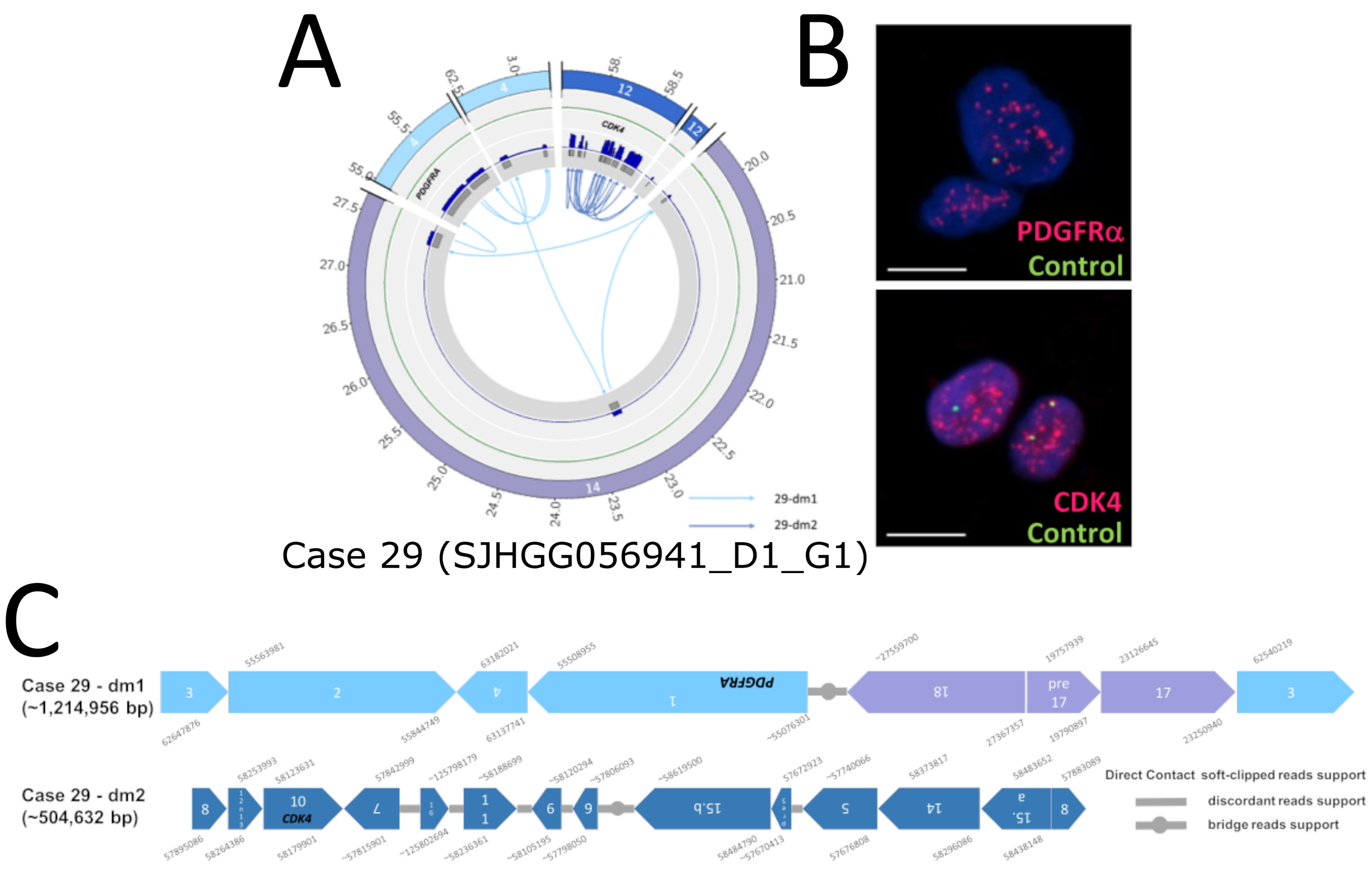

Supplementary Figure 8. Example of intra- and inter-chromosomal rearrangements leading to eccDNA structures. A) Circos plot illustrating the intra-chromosomal arrangement of chromosome 14 involving *CDK4* (dm2) and inter-chromosomal rearrangements of chromosomes 4 and 14 involving *PDGFRA* (dm1). The innermost circle shows highly amplified CNA segments, determined by comparing their coverage in the tumor sample (blue circle) with that in the paired germline sample (green circle). The outermost circle shows the chromosomes involved. B) Two-color FISH experiment confirming gene amplification. Upper panel shows *PDGFRA*, and the lower panel shows *CDK4* amplification with diffuse punctate staining relative to the control centromeric DNA in each cell, suggesting the presence of multiple double minutes. The double-minute pattern is typical of episomal amplification. Confirmation of double minutes by other methods, such as creating a metaphase spread, was not possible because samples tested by FISH (and the only samples available for confirmation) were paraffin-embedded samples for which cultured cells were not available. For FISH assays, targets were probed N=1 time per target per case with proper controls counting 200 cells for each target assayed. Representative images are shown. C) Predicted structures of the eccDNA dm1 and dm2 showing the directions of each of the joined CNA segments and their break point positions. “Bridge reads support” means SVs identified through common discordant reads support between two segment boundaries. SVs, structural variants. CNA, copy number alterations; FISH, fluorescence in situ hybridization; CNA, copy number alterations; FISH, fluorescence in situ hybridization.

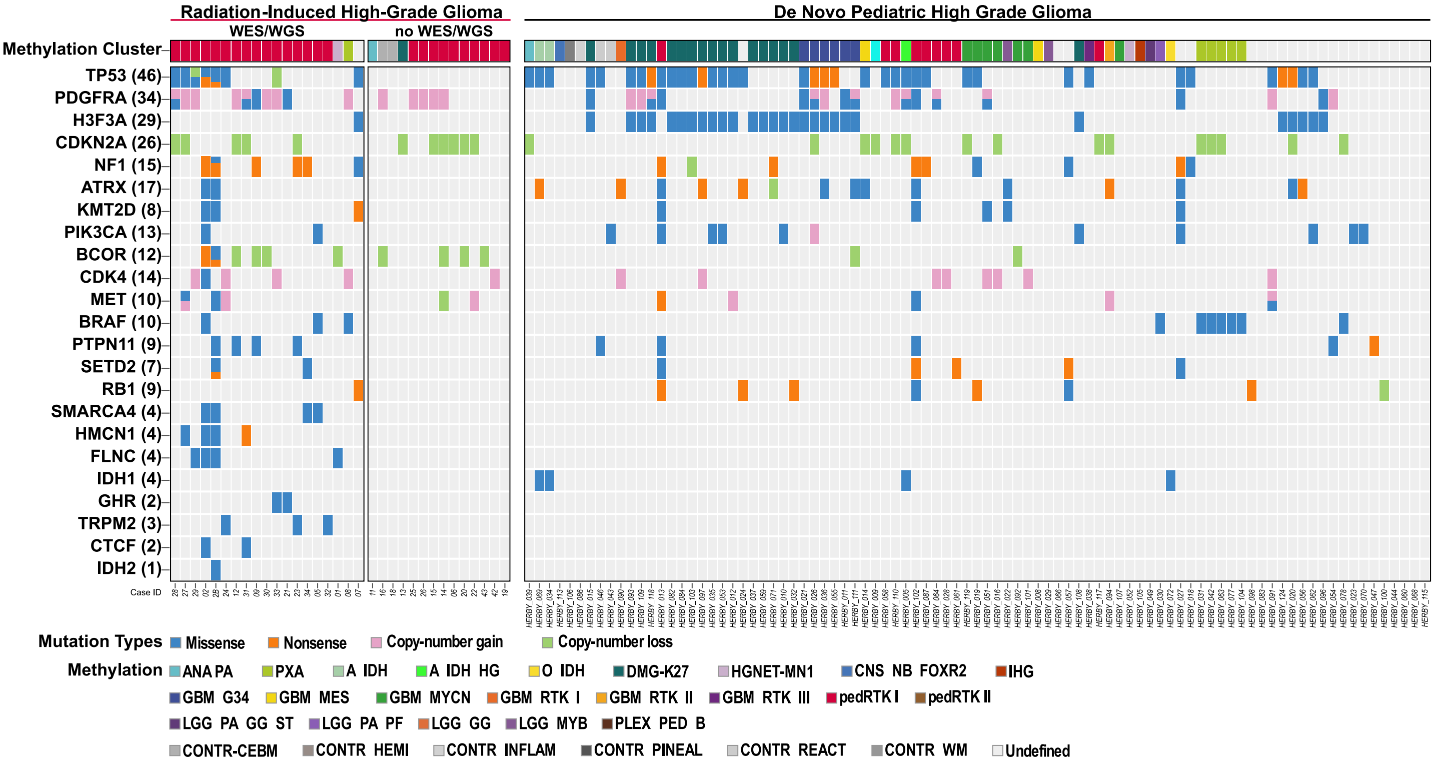

Supplementary Figure 9. Recurrent molecular alterations in RIG compared to *de novo* pHGG (HERBY dataset). Oncoprint describing the consensus methylation group, tier1 mutations, and genes affected by copy-number gain/loss in RIG and *de novo* pHGG (HERBY). RIG, radiation-induced high-grade gliomas; pHGG, pediatric high-grade gliomas.

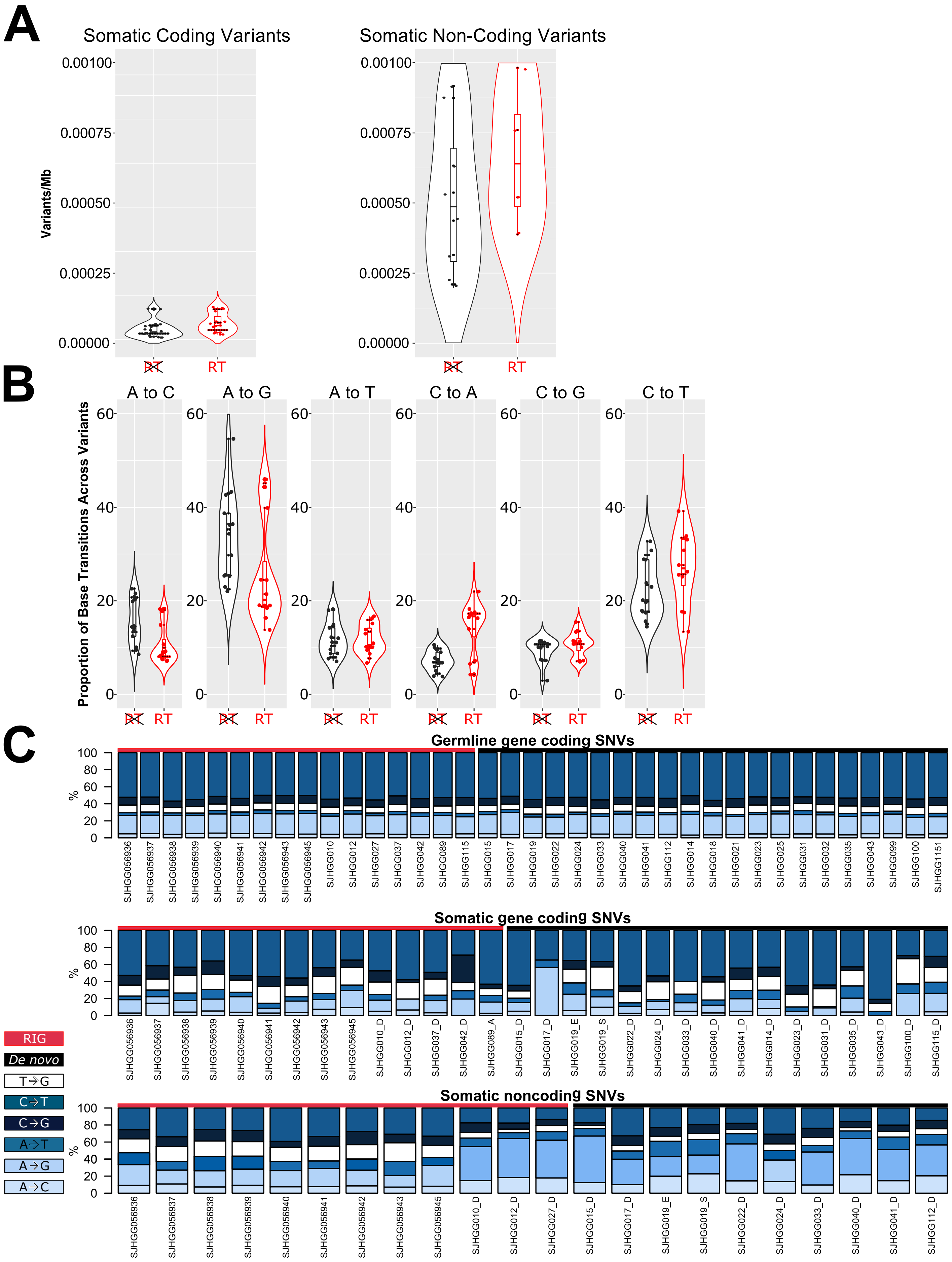
A
Somatic Coding Variants
Somatic Non-Coding Variants
Variants/Mb
0.00100
0.00075
0.00050
0.00025
0.00000
RT
RT
B
A to C
A to G
A to T
C to A
C to G
C to T
Proportion of Base Transitions Across Variants
60
40
20
0
C
Germline gene coding SNVs
%
100
80
60
40
20
0
SJHGG056936
SJHGG056937
SJHGG056938
SJHGG056939
SJHGG056940
SJHGG056941
SJHGG056942
SJHGG056943
SJHGG056945
SJHGG010
SJHGG012
SJHGG027
SJHGG037
SJHGG042
SJHGG089
SJHGG115
SJHGG015
SJHGG017
SJHGG019
SJHGG022
SJHGG024
SJHGG033
SJHGG040
SJHGG041
SJHGG112
SJHGG014
SJHGG018
SJHGG021
SJHGG023
SJHGG025
SJHGG031
SJHGG032
SJHGG035
SJHGG043
SJHGG099
SJHGG100
SJHGG1151
Somatic gene coding SNVs
SJHGG056936
SJHGG056937
SJHGG056938
SJHGG056939
SJHGG056940
SJHGG056941
SJHGG056942
SJHGG056943
SJHGG056945
SJHGG010_D
SJHGG012_D
SJHGG037_D
SJHGG042_D
SJHGG089_A
SJHGG015_D
SJHGG017_D
SJHGG019_E
SJHGG019_S
SJHGG022_D
SJHGG024_D
SJHGG033_D
SJHGG040_D
SJHGG041_D
SJHGG014_D
SJHGG023_D
SJHGG031_D
SJHGG035_D
SJHGG043_D
SJHGG100_D
SJHGG115_D
RIG
De novo
T→G
C→T
C→G
A→T
A→G
A→C
Somatic noncoding SNVs
SJHGG056936
SJHGG056937
SJHGG056938
SJHGG056939
SJHGG056940
SJHGG056941
SJHGG056942
SJHGG056943
SJHGG056945
SJHGG010_D
SJHGG012_D
SJHGG027_D
SJHGG015_D
SJHGG017_D
SJHGG019_E
SJHGG019_S
SJHGG022_D
SJHGG024_D
SJHGG033_D
SJHGG040_D
SJHGG041_D
SJHGG112_D

Supplementary Figure 10. Somatic variants in RIG relative to *de novo* pHGG (Wu G reference cohort) stratified by base transition type among cases with available WGS. A) Total number of somatic coding, and somatic non-coding variants per Mb stratified by *de novo* pHGG (Wu cohort, labeled with "RT" that has been x'd out) (0.000700 ± 0.000245 (range) variants/Mb,) vs. RIG (labeled "RT") (median 0.000784 ± 0.000286 (range) variants/Mb, p=0.031) (Student's two-sample, two-sided t-test). B) Proportion of base transitions in somatic non-coding variants per Mb stratified by *de novo* pHGG vs. RIG. Relative frequencies of somatic non-coding base transitions were decreased for A to C (median 14.4% ± 4.91 vs. 8.8% ± 4.1, p=0.007), and A to G (34.3% ± 9.67 vs. 20.2% ± 11.3, p=0.03) in RIG relative to pHGG, but increased for C to A transitions (6.79% ± 2.2 vs. 16.7% ± 5.47, p=0.004) (Student's two-sample two-sided t-test). C) The relative frequency of base transitions in germline coding SNVs, somatic coding SNVs, and somatic non-noncoding SNVs. For violin plots, box shows median and interquartile range, whiskers show 95% confidence interval, total length of violin represents range, and width of violin shows frequency. RIG, radiation-induced high-grade glioma; pHGG, pediatric high-grade glioma; SNV, single nucleotide variation; WFS, whole genome sequencing., The p-values in panels A and B did not involve multiple comparisons so no multiple comparison corrections were performed. In panels A and B the median is represented by the central bar while the 1st and 3rd quartile are represented by the flanking bounds of the box, the range of the values is indicated by the bounds of the perpendicular line for each box and the solitary points represent outliers. The smoothed distribution is represented by the contour of the violin.

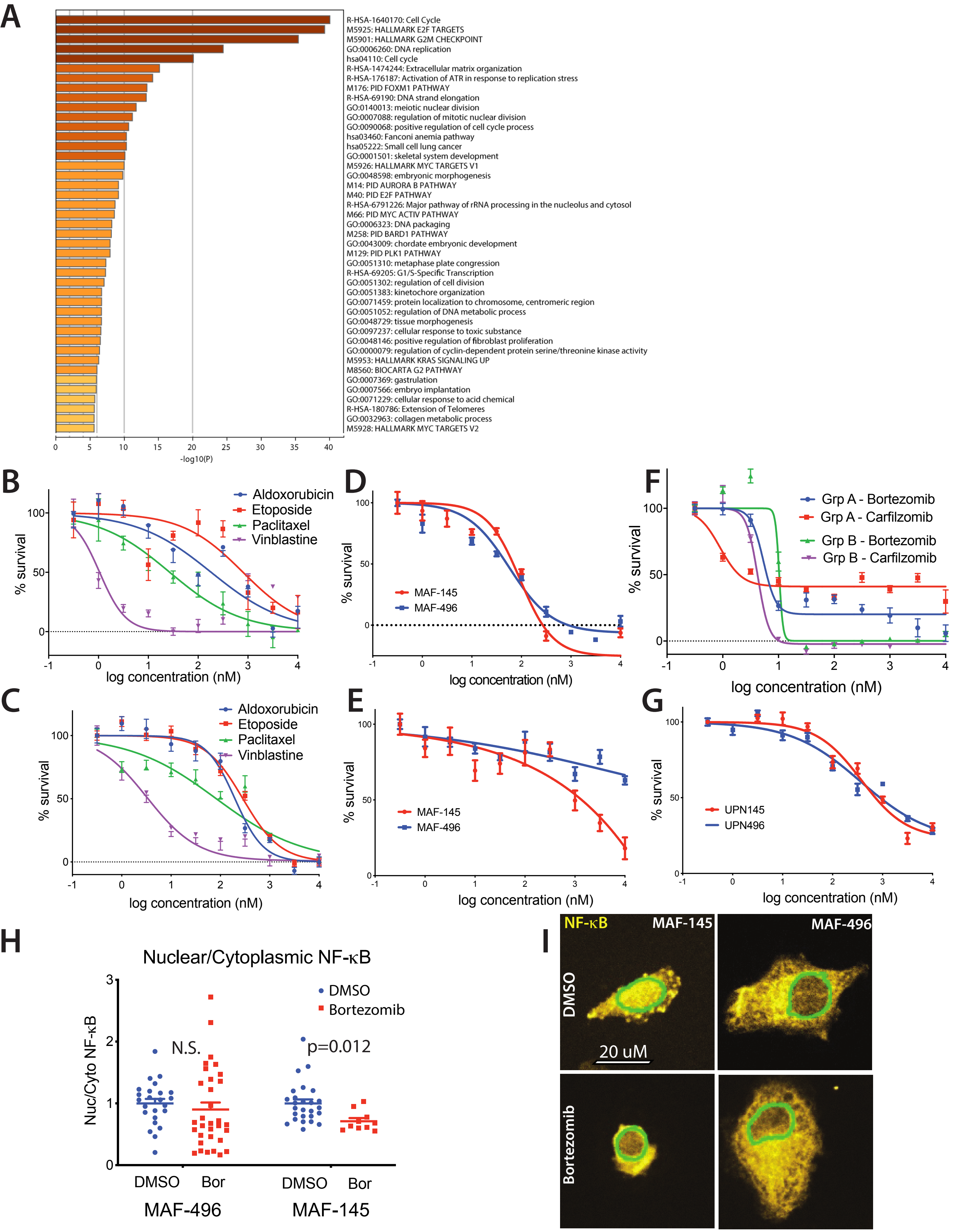
A
R-HSA-1640170: Cell Cycle
M5925: HALLMARK E2F TARGETS
M5901: HALLMARK G2M CHECKPOINT
GO:0006260: DNA replication
hsa04110: Cell cycle
R-HSA-1474244: Extracellular matrix organization
R-HSA-176187: Activation of ATR in response to replication stress
M176: PID FOXM1 PATHWAY
R-HSA-69190: DNA strand elongation
GO:0140013: meiotic nuclear division
GO:0007088: regulation of mitotic nuclear division
GO:0090068: positive regulation of cell cycle process
hsa03460: Fanconi anemia pathway
hsa05222: Small cell lung cancer
GO:0001501: skeletal system development
M5926: HALLMARK MYC TARGETS V1
GO:0048598: embryonic morphogenesis
M14: PID AURORA B PATHWAY
M40: PID E2F PATHWAY
R-HSA-6791226: Major pathway of rRNA processing in the nucleolus and cytosol
M66: PID MYC ACTIV PATHWAY
GO:0006323: DNA packaging
M258: PID BARD1 PATHWAY
GO:0043009: chordate embryonic development
M129: PID PLK1 PATHWAY
GO:0051310: metaphase plate congression
R-HSA-69205: G1/S-Specific Transcription
GO:0051302: regulation of cell division
GO:0051383: kinetochore organization
GO:0071459: protein localization to chromosome, centromeric region
GO:0051052: regulation of DNA metabolic process
GO:0048729: tissue morphogenesis
GO:0097237: cellular response to toxic substance
GO:0048146: positive regulation of fibroblast proliferation
GO:0000079: regulation of cyclin-dependent protein serine/threonine kinase activity
M5953: HALLMARK KRAS SIGNALING UP
M8560: BIOCARTA G2 PATHWAY
GO:0007369: gastrulation
GO:0007566: embryo implantation
GO:0071229: cellular response to acid chemical
R-HSA-180786: Extension of Telomeres
GO:0032963: collagen metabolic process
M5928: HALLMARK MYC TARGETS V2
0 5 10 15 20 25 30 35 40
-log10(P)
B
Aldoxorubicin
Etoposide
Paclitaxel
Vinblastine
% survival
log concentration (nM)
C
Aldoxorubicin
Etoposide
Paclitaxel
Vinblastine
% survival
log concentration (nM)
D
MAF-145
MAF-496
% survival
log concentration (nM)
E
MAF-145
MAF-496
% survival
log concentration (nM)
F
Grp A - Bortezomib
Grp A - Carfilzomib
Grp B - Bortezomib
Grp B - Carfilzomib
% survival
log concentration (nM)
G
UPN145
UPN496
% survival
log concentration (nM)
H
Nuclear/Cytoplasmic NF-κB
DMSO
Bortezomib
Nuc/Cyto NF-κB
N.S.
p=0.012
DMSO
Bor
DMSO
Bor
MAF-496
MAF-145
I
NF-κB
MAF-145
MAF-496
DMSO
Bortezomib
20 uM

Supplementary Figure 11. RIG *in vitro* sensitivities by expression profile. A) MetaScape analysis of 2,000 genes upregulated in RIG tumors versus normal cortical tissue. (B–G) *In vitro* drug screen results in two primary cell lines derived from RIG tumors: MAF-145 (gene expression RIG Group B) and MAF-496 (gene expression RIG Group A). Drugs for validation included two DNA-damaging agents (aldoxorubicin and etoposide), two microtubule agents (paclitaxel and vinblastine), three proteasome inhibitors (bortezomib, carfilzomib, and marizomib), and two kinase inhibitors (the multi-TKI inhibitor sunitinib and the MEK inhibitor trametinib). B) MTS assay results for indicated drugs in MAF-496 (IC_{50} values listed in Fig. 7D). C) MTS results for indicated drugs in MAF-145 (IC_{50} values listed in Fig. 7D). D) MTS results for trametinib. E) MTS results for sunitinib. F) MTS assay results for proteasome inhibitors bortezomib and carfilzomib in MAF-145 (Grp B) and MAF-496 (Grp B) cell lines. G) MTS results for marizomib in MAF-145 and MAF-496. H) NF-κB levels in Group A (MAF-496) and B (MAF-145) RIG cell lines treated with vehicle (0.1% DMSO) or bortezomib (100nM) for 16h. Nuclear levels are mean NF-κB expression computed in ImageJ. Nuclear extent was determined using DAPI staining and Analyze Cells tool in ImageJ. Cytoplasmic levels are calculated as total cell integrated density × total cell area – nuclear integrated density × nuclear area)/(total cell area – nuclear area). Confocal imaging was performed as described in the Methods. Mean and SEM shown. I) Immunofluorescence staining example showing NF-κB nuclear localization in vehicle versus bortezomib-treated Group A (MAF-496) and Group B (MAF-145) RIG cell lines. MTS, MetaScape analysis; RIG, radiation-induced high-grade glioma; SEM, standard error of the mean. The experiment described in panels h and i was performed one time due to material availability; multiple cells were interrogated to present the results, as shown in panel h. The p-values for panel a are calculated within Metascape using a hypergeometric distribution to estimate the probability of obtaining *M* genes in a particular pathway from a larger pool of *N* genes. Details are available at: http://metascape.org/blog/?p=122. In panel h, Student's t-test (two-sample, two-sided) was used to compute the *p*-value; multiple comparisons were not involved in the analysis and no correction for multiple comparisons was performed.

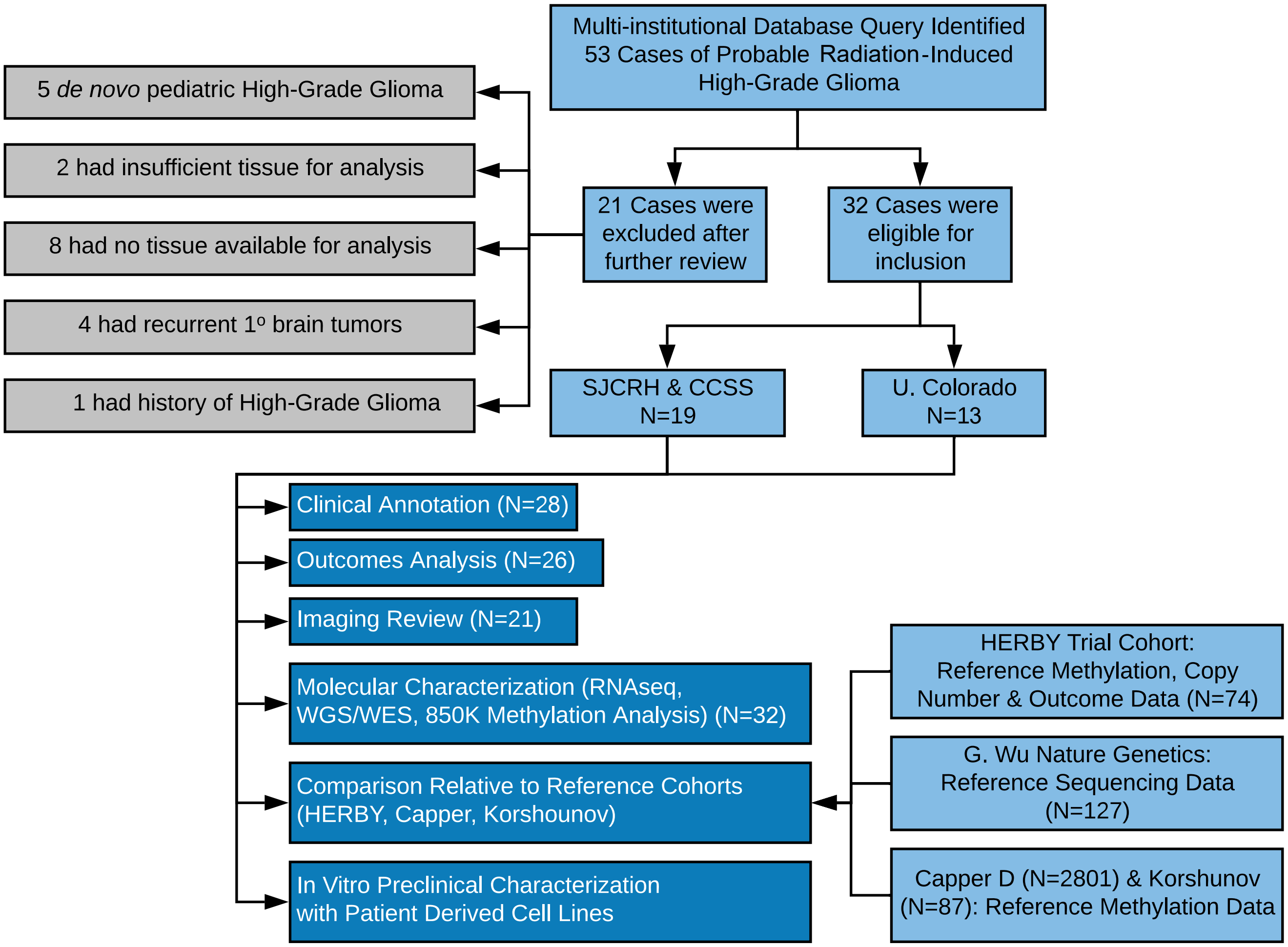

Supplementary Figure 12. Study consort diagram. CCSS, Childhood Cancer Survivor Study; SJCRH, St. Jude Children's Research Hospital; U. Colorado, University of Colorado; WGS, whole-genome sequencing; WES, whole-exome sequencing; RNA-seq, RNA sequencing.

Supplementary Table 1. Patient, Treatment, and Initial and RIG Disease Characteristics

Patient Characteristics	Subgroup	N	Median (range) or Frequency
**Age at Initial Diagnosis**	years	27	7 (0.16-19)
**Initial Diagnosis**	ALL	10	31%
	Glial tumor, other	3	9%
	Ganglioglioma	1	3%
	Ependymoma	2	6%
	Burkitt Lymphoma	1	3%
	Craniopharyngioma	1	3%
	Germinoma	2	6%
	Medulloblastoma	12	38%
**Initial Radiotherapy Indication**	Adjuvant	12	38%
	Salvage	9	28%
	Definitive	1	3%
	Unknown	13	40%
**Initial Radiotherapy Dose**	Initial Field	9	23.4 (23.4-36)
	Boost	9	51 Gy (12-59.4 Gy)
**Initial Radiotherapy Extent**	CSI	9	28%
	Cranial	6	18%
	Focal	9	28%
	TBI	1	3%
	Unknown	7	22%
**Age at RIG Diagnosis**	years	27	14 (4-26)
**RIG Location Relative**	Unknown	10	31%
**to Radiation Fields**	In field, non-Rx Dose	8	25%
	In field, Rx Dose	17	53%
**RIG Histology**	High-Grade Glioma	11	34%
	Anaplastic Astrocytoma	3	9%
	Glioblastoma Multiforme	18	56%
**RIG Anatomic Location**	Cerebellum	8	25%
	Cerebrum	12	38%
	Spine	1	3%
	Cerebrum+Cerebellum	1	3%
	Brainstem+Cerebellum	2	6%
	Unknown	11	34%

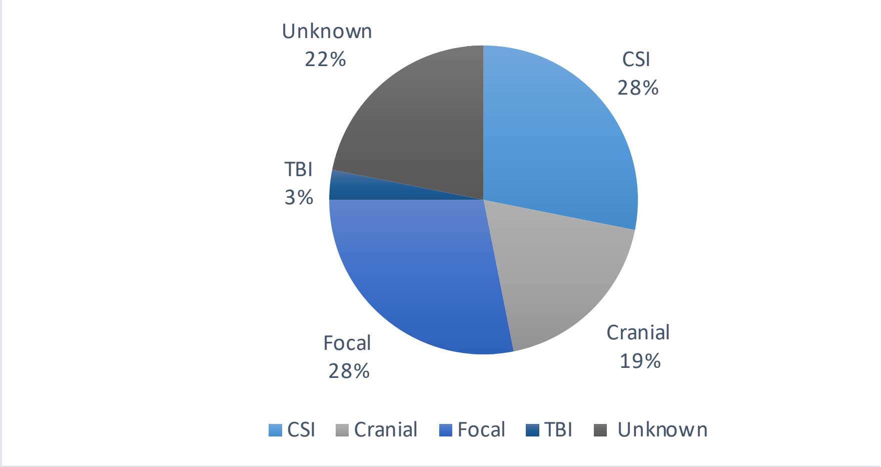

Supplementary Table 2. RIG Treatment Characteristics and Outcomes

Treatment Characteristic	Subset	N	Median (range) or Frequency
**RIG Treatment**	Resection	2	6.0%
	RT + Surg	1	3.0%
	Chemo	4	13.0%
	RT	11	34.0%
	Unknown	22	69.0%
**Second Radiotherapy Dose**	Known	8	54 Gy (30-59.4)
	Unknown	2	6.0%
**Outcome**	Alive	0	0.0%
	Dead	26	81.0%
	[illegible]	[illegible]	[illegible]

Supplementary Table 3. Methylation-based Classification Probabilities - Reference Dataset (Korshunov A and Capper D)

SentrixID	ID	Class Call	loss_1p	gain_1q	chr13	chr14	PDGFRA	CDK4	CDKN2A
9969477031_R04C02	Capper et al (SAMPLE 2152)	GBM-MID	LOSS	GAIN	WT	WT	AMP	WT	WT
3999834021_R04C02	Capper et al (SAMPLE 2475)	GBM-MID	WT	WT	WT	LOSS	AMP	WT	WT
9969477086_R01C01	Capper et al (SAMPLE 2199)	GBM-MID	WT	WT	PARTIAL LOSS	PARTIAL LOSS	AMP	WT	DEL
9855358032_R03C01	Capper et al (SAMPLE 2255)	GBM-MID	WT	WT	LOSS	WT	AMP	WT	DEL
9878820018_R01C01	Capper et al (SAMPLE 2292)	GBM-MID	WT	WT	WT	WT	WT	WT	DEL
3999834021_R05C01	Capper et al (SAMPLE 2477)	GBM-MID	WT	WT	WT	WT	WT	WT	WT
9969477063_R02C02	Capper et al (SAMPLE 2171)	GBM-MID	LOSS	WT	WT	WT	AMP	WT	DEL
9374341006_R04C02	Capper et al (SAMPLE 1214)	GBM-MID	WT	PARTIAL GAIN	PARTIAL_LOSS	PARTIAL_LOSS	AMP	WT	DEL
3999834105_R02C01	Capper et al (SAMPLE 2572)	GBM-MID	WT	WT	LOSS	WT	AMP	WT	DEL
3999543050_R02C02	Capper et al (SAMPLE 2597)	GBM-MID	PARTIAL_LOSS	PARTIAL GAIN	WT	PARTIAL_LOSS	AMP	WT	DEL
8622007063_R01C01	Capper et al (SAMPLE 636)	GBM-MID	WT	WT	PARTIAL LOSS	WT	WT	WT	DEL
9741950100_R04C02	Capper et al (SAMPLE 1819)	GBM-MID	LOSS	WT	WT	PARTIAL LOSS	AMP	WT	WT
9741950099_R02C01	Capper et al (SAMPLE 1801)	GBM-MID	LOSS	WT	LOSS	LOSS	AMP	WT	DEL
9533774097_R01C01	Capper et al (SAMPLE 1995)	GBM-MID	LOSS	WT	LOSS	WT	AMP	WT	DEL
201490030243_R04C01	Korshunov A et al (201490030243_R04C01)	pedRTK I	WT	WT	PARTIAL LOSS	PARTIAL LOSS	WT	WT	DEL
9340996014_R03C01	Korshunov A et al (9340996014_R03C01)	pedRTK I	WT	WT	WT	WT	WT	WT	WT
10006823133_R02C01	Korshunov A et al (10006823133_R02C01)	pedRTK I	WT	WT	LOSS	WT	WT	WT	DEL
5684819014_R04C01	Korshunov A et al (5684819014_R04C01)	pedRTK I	WT	WT	WT	WT	WT	WT	DEL
9406921073_R03C02	Korshunov A et al (9406921073_R03C02)	pedRTK I	WT	WT	WT	WT	WT	WT	WT
9741950109_R05C01	Korshunov A et al (9741950109_R05C01)	pedRTK I	WT	PARTIAL GAIN	WT	WT	WT	WT	WT
9444375018_R02C01	Korshunov A et al (9444375018_R02C01)	pedRTK I	WT	WT	WT	WT	WT	WT	DEL
6164621081_R02C01	Korshunov A et al (6164621081_R02C01)	pedRTK I	WT	WT	WT	WT	WT	WT	WT
9340996032_R03C02	Korshunov A et al (9340996032_R03C02)	pedRTK I	WT	WT	PARTIAL LOSS	WT	WT	WT	WT
200277210073_R06C02	Korshunov A et al (200277210073_R06C02)	pedRTK I	LOSS	GAIN	LOSS	WT	AMP	WT	WT
200930770009_R03C01	Korshunov A et al (200930770009_R03C01)	pedRTK I	PARTIAL_LOSS	WT	PARTIAL LOSS	LOSS	AMP	WT	WT
200723300044_R03C01	Korshunov A et al (200723300044_R03C01)	pedRTK I	PARTIAL_LOSS	PARTIAL GAIN	WT	WT	AMP	WT	DEL
201465930013_R05C01	Korshunov A et al (201465930013_R05C01)	pedRTK I	PARTIAL_LOSS	WT	LOSS	WT	WT	WT	DEL
9761749097_R03C02	Korshunov A et al (9761749097_R03C02)	pedRTK I	PARTIAL_LOSS	PARTIAL GAIN	WT	WT	WT	WT	WT
9741950099_R06C01	Korshunov A et al (9741950099_R06C01)	pedRTK I	LOSS	PARTIAL GAIN	LOSS	PARTIAL LOSS	AMP	WT	WT
9741950102_R04C01	Korshunov A et al (9741950102_R04C01)	pedRTK I	PARTIAL_LOSS	PARTIAL GAIN	WT	LOSS	WT	WT	WT

Supplementary Table 4. Comparison of genes presented in Fig. 6D from GO_DNA_REPAIR geneset in Group B vs. Group A

Common_name	B/A	P-val	Grp A Mean	SEM	Grp B Mean	SEM2
GTF2H2	0.44	0.00	23.79	2.86	10.52	1.28
PAPD7	0.27	0.00	3.31	0.24	0.88	0.42
TTC5	0.70	0.00	18.79	0.90	13.12	0.69
GTF2H2C	0.62	0.01	13.69	1.75	8.42	0.92
WDR48	0.69	0.01	53.89	5.60	37.13	3.37
LIG3	0.71	0.01	33.02	1.90	23.51	2.41
MLH1	0.75	0.02	57.12	3.42	43.00	1.34
POLD3	1.35	0.02	11.99	1.34	16.25	1.16
UPF1	1.29	0.02	28.65	2.73	37.05	2.50
MMS19	1.51	0.02	48.57	4.28	73.31	7.19
MSH6	0.71	0.03	61.79	7.15	43.69	2.10
Id	0.70	0.03	18.71	2.30	13.16	1.36
FANCE	0.78	0.03	11.89	0.64	9.30	0.94
SLX4	0.67	0.03	8.58	1.16	5.74	0.41
TAOK3	0.67	0.04	73.67	9.30	49.07	6.61
USP7	0.84	0.04	188.87	13.46	158.99	10.95
ERCC4	0.74	0.04	7.91	0.99	5.81	0.59
ZSWIM7	0.55	0.04	93.44	20.89	51.28	4.00
RFWD3	0.72	0.04	29.98	3.34	21.56	1.74
RMI2	0.70	0.04	22.44	3.22	15.68	1.04
UBE2T	0.55	0.04	135.57	27.15	74.65	8.58
XPC	0.76	0.05	34.95	3.55	26.58	1.46
PMS2	0.82	0.05	12.22	1.29	10.02	0.45
RAD51D	0.77	0.05	17.53	0.74	13.52	1.58
TP53	0.60	0.05	154.36	32.40	92.56	20.67

**Mean**	0.76	
**SEM**	0.02	
**t-statistic**	10.48	
**p-value vs. Expected val**	0.00	Student's t Test
