# Supplementary material for: Comprehensive molecular characterization of pediatric radiation-induced high-grade glioma
Source: Nat Commun. 2021 Sep 20;12:5531. doi: 10.1038/s41467-021-25709-x (PMC8452624; doi:10.1038/s41467-021-25709-x)
Supplement: Supplementary file 3 — Description of Additional Supplementary Files [file 41467_2021_25709_MOESM3_ESM.docx]

**Description of Additional Supplementary Files**

**Supplementary Data 1.** Summary of methylation-based classification and genetic alterations, germline status, gene expression-based classification and chromothripsis findings. Data columns include summary data (Cols. A–F); hypermutator status (Col. G); antecedent tumor treatment (Cols. H–L); RIG characteristics, including age, latency, RIG location and histology and survival (Cols. M–W); samples with RNA-seq, WES, WGS, and methylation data (Cols. Z – AD), methylation classification (Cols. AE-AI); focal and large-scale CNVs (Cols. AJ – AT); gene expression subgroup (Col. AU); and presence of chromothripsis (Col. AV). Key: 0, No; 1, yes; Gy, Gray; WBRT, whole brain radiotherapy; CSI, craniospinal radiation, IVth, fourth; R, right; TBI, total body irradiation; TTP, time to progression; TTD2, time to death from antecedent cancer; TTD, time to death from RIG diagnosis. Corresponding methylation cluster assignments are shown in columns German_Group_Top, GERMAN, TSNE, HCLUST, and CONSENSUS. Frequent copy number alterations are shown in columns AJ-AS. MGMT methylation status is shown in column AT. Expression group is defined in column AU.

**Supplementary Data 2.** Methylation-based Classification Probabilities - German-Classifier Results_20181214. Methylation-based classification probabilities derived from case submission to the German (DKFZ) classifier as of December 14, 2018. Dark red shading indicates a higher probability of group assignment.

**Supplementary Data 3.** Comparison of RIG and Primary Pediatric High-Grade Glioma Methylation Class. Comparison of the proportion of methylation classes in each cohort (RIG and *de novo* pHGG (HERBY). The odds of assigning a RIG to pedRTK I was 29.2 (9.15–92.92, *p*<0.0001) relative to a *de novo* pHGG from the HERBY cohort.

**Supplementary Data 4.** RIG structural variations identified in 10 WGS cases.

**Supplementary Data 5.** RIG copy-number alteration segments identified in 10 WGS cases

**Supplementary Data 6.** Comparison of RIG and Primary Pediatric High-Grade Gliomas from the HERBY cohort. Comparison of frequent SNVs and CNVs across RIG and *de novo* pHGG from the HERBY cohort, stratified by methylation group. FET, Fisher’s Exact test; Chi, Chi-square test; wt, wild type; amp, amplification; mut, mutant.

**Supplementary Data 7.** Statistical analysis of chromothripsis events in 12 RIGs. The total number structural variants are indicated in column C. The affected chromosome(s) are indicated in column D. Intra-chromosomal structural variants are indicated in column E. The presence or absence of oscillating copy number alterations are indicated in column F. The presence or absence of clustering of chromosomal breakpoints is indicated in column G. The probability of randomness of the segment order is indicated in column H. The chromothripsis criteria call based on the presence of at least 2 positive criteria is listed as yes or no in column J. The consensus call after manual review is indicated in column K. The proportion of cases with chromothripsis in the non-brainstem (NBS), DIPG, and DIPG+NBS *de novo* pHGG 2014 Nature Genetics cohort relative to the RIG cohort is shown in the table in rows 29-36. Corresponding one-sided fisher’s Exact tests comparing each group to RIG are shown in column E.

**Supplementary Data 8.** Sequence mutations (Tier1) identified for 5 non-hypermutator WES cases without matched germline data

**Supplementary Data 9.** Sequence mutations (Tier1) identified for 9 non-hypermutator WGS cases with matched germline data

**Supplementary Data 10.** Germline variants for 9 non-hypermutator WGS cases

**Supplementary Data 11.** Methylation-based Classification Probabilities - HERBY Dataset. Methylation-based classification probabilities for the *de novo* pHGG HERBY dataset and corresponding high-frequency copy-number alterations of interest for comparison to RIG. Survival data are indicated in column BB and BC.

**Supplementary Data 12.** Fusion genes identified in RNAseq samples. Type of fusion is indicated in column L. The frame column U is defined as follows: *in frame*: all predicted cDNA products are in frame; *likely in frame:* some predicted cDNA products are in frame, some are out of frame; *out of frame*: all predicted cDNA products are out of frame.

**Supplementary Data 13.** Output of Metascape analysis in RIG vs. *de novo* GBM microarray data from Children’s Hospital Colorado (CHCO).

**Supplementary Data 14.** GSEA results in RIG vs. *de novo* GBM (CHCO) for GO_All geneset database, with all genesets shown. GO_All geneset is found and described in detail at: msigdb/gmt/c5.all.v7.2.symbols.gmt

**Supplementary Data 15.** RIG Group B vs. A geneset enrichment analysis results grouped by category as shown in Figure 6A.

**Supplementary Data 16.** *In Silico* Drug Screen Results. GSEA results for RIG versus normal cortex and RIG versus *de novo* GBM (CHCO).

**Supplementary Data 17.** *In Vitro* Drug Screen Results. Screening results for FDA-approved anti-cancer agents in MAF-145 and MAF-496 cell lines.

**Supplementary Data 18.** Focal copy number alterations by gene. Amplifications are shown in graded shades of red while deletions are shown in graded shades of blue. q-values were determined using GISTIC (v 2.0.23).

**Supplementary Data 19.** Broad copy number alterations by gene. Amplifications are shown in graded shades of red while deletions are shown in graded shades of blue. q-values were determined using GISTIC (v 2.0.23).

**Supplementary Data 20.** Broad copy number alterations by chromosome arm. Amplifications are shown in graded shades of red while deletions are shown in graded shades of blue. The amplification and deletion frequency, frequency score, z-score, and q-value were determined using GISTIC (v 2.0.23) for the RIG and HERBY high grade glioma group, respectively. Tabulated comparisons are shown in Supplementary Data 6.
