# Supplementary material for: Comprehensive molecular characterization of pediatric radiation-induced high-grade glioma
Source: Nat Commun. 2021 Sep 20;12:5531. doi: 10.1038/s41467-021-25709-x (PMC8452624; doi:10.1038/s41467-021-25709-x)
Supplement: Supplementary file 24 — Reporting Summary [file 41467_2021_25709_MOESM24_ESM.pdf]

nature research

Corresponding author(s): Adam Green and Brent Orr

Last updated by author(s): Apr 20, 2021

Reporting Summary

Nature Research wishes to improve the reproducibility of the work that we publish. This form provides structure for consistency and transparency in reporting. For further information on Nature Research policies, see our Editorial Policies and the Editorial Policy Checklist.

Statistics

For all statistical analyses, confirm that the following items are present in the figure legend, table legend, main text, or Methods section.

n/a	Confirmed	
☐	☒	The exact sample size (*n*) for each experimental group/condition, given as a discrete number and unit of measurement
☐	☒	A statement on whether measurements were taken from distinct samples or whether the same sample was measured repeatedly
☐	☒	The statistical test(s) used AND whether they are one- or two-sided<br>*Only common tests should be described solely by name; describe more complex techniques in the Methods section.*
☐	☒	A description of all covariates tested
☐	☒	A description of any assumptions or corrections, such as tests of normality and adjustment for multiple comparisons
☐	☒	A full description of the statistical parameters including central tendency (e.g. means) or other basic estimates (e.g. regression coefficient) AND variation (e.g. standard deviation) or associated estimates of uncertainty (e.g. confidence intervals)
☐	☒	For null hypothesis testing, the test statistic (e.g. *F*, *t*, *r*) with confidence intervals, effect sizes, degrees of freedom and *P* value noted<br>*Give P values as exact values whenever suitable.*
☒	☐	For Bayesian analysis, information on the choice of priors and Markov chain Monte Carlo settings
☒	☐	For hierarchical and complex designs, identification of the appropriate level for tests and full reporting of outcomes
☒	☐	Estimates of effect sizes (e.g. Cohen's *d*, Pearson's *r*), indicating how they were calculated

Our web collection on statistics for biologists contains articles on many of the points above.

Software and code

Policy information about availability of computer code

Data collection	BGI Americas/BGISEQ-500 (whole genome sequencing); Illumina HiSeq 4000 (whole exome sequencing); RNA-Seq); Illumina HiSeq 2500 (RNA-Seq data); Affymetrix HG133 (microarray-based gene expression); Illumina InfiniumEPIC and HumanMethylation450 microarrays (DNA methylation).
Data analysis	Rstudio Version 1.1.463 (patient- and sample-level statistical analyses), open source statistical programming language R (R Core Team, 2016, DNA methylation data analysis, RNA-seq analysis), www.molecularneuropathology.org (supervised DNA methylation analysis), CICERO (fusion gene detection in RNA-seq), Metascape 3.5 (differentially enriched pathways in microarray data), GSEA 4.0.x (Broad Institute), MSigDB 7.0 and 7.1 (geneset database for gene expression analysis); and IPA Spring 2017 Release (Qiagen) (RNA-seq subgrouping and expression patterns), Bambino/Medal Ceremony (WES variant calls/annotation); GISTIC (v 2.0.23)

For manuscripts utilizing custom algorithms or software that are central to the research but not yet described in published literature, software must be made available to editors and reviewers. We strongly encourage code deposition in a community repository (e.g. GitHub). See the Nature Research guidelines for submitting code & software for further information.

Data

Policy information about availability of data

All manuscripts must include a data availability statement. This statement should provide the following information, where applicable:

- Accession codes, unique identifiers, or web links for publicly available datasets
- A list of figures that have associated raw data
- A description of any restrictions on data availability

This information has been added to the data availability statement in the manuscript.

Field-specific reporting

Please select the one below that is the best fit for your research. If you are not sure, read the appropriate sections before making your selection.

☒ Life sciences ☐ Behavioural & social sciences ☐ Ecological, evolutionary & environmental sciences

For a reference copy of the document with all sections, see nature.com/documents/nr-reporting-summary-flat.pdf

Life sciences study design

All studies must disclose on these points even when the disclosure is negative.

Sample size	The sample size of total tumor and matched blood samples was based on availability between the participating institutions; sample sizes for each tumor and blood sample analysis was also based on feasibility from available specimen materials (FFPE, frozen, etc).
Data exclusions	20 cases were excluded after review, as detailed in Supplemental Figure 1. Cases were excluded if upon further review there was insufficient tissue for further analyses, not consistent with a treatment induced glioma, had the same histology as the incident cancer diagnosis or had a history of prior high grade glioma.
Replication	The in vitro drug screen in MAF145 was performed in duplicate; the in vitro drug screen in MAF496 was performed one time due to inability to expand the cells in culture; Individual drug validations were conducted in triplicate; staining for NF-KB was conducted in duplicate in each cell line.
Randomization	No allocation of samples between control and experimental groups was performed in this study.
Blinding	No allocation of samples was performed so blinding is irrelevant to the study.

Reporting for specific materials, systems and methods

We require information from authors about some types of materials, experimental systems and methods used in many studies. Here, indicate whether each material, system or method listed is relevant to your study. If you are not sure if a list item applies to your research, read the appropriate section before selecting a response.

Materials & experimental systems

n/a	Involved in the study	
☐	☒	Antibodies
☐	☒	Eukaryotic cell lines
☒	☐	Palaeontology and archaeology
☒	☐	Animals and other organisms
☐	☒	Human research participants
☐	☒	Clinical data
☒	☐	Dual use research of concern

Methods

n/a	Involved in the study	
☒	☐	ChIP-seq
☒	☐	Flow cytometry
☒	☐	MRI-based neuroimaging

Antibodies

Antibodies used	NF-kB antibody - Cell Signaling #6956
Validation	In prior studies, we verified that this antibody does not produce signal in cells where NF-kB has been suppressed and produces signal in cells where NF-kB has been activated. These studies were done in connection with another project performed by the Green lab.

Eukaryotic cell lines

Policy information about cell lines

Cell line source(s)	Two cell lines developed from primary tumor samples were used.
Authentication	Cells were disaggregated from primary tumor at the time of tumor collection, expanded briefly and frozen. Cells were not passaged. Authentication was based on chain of custody. In performing the experiments for this article, we were unable to passage either cell line; therefore, further authentication was not conducted.
Mycoplasma contamination	Cell lines were not tested for mycoplasma due to the lack of ability to passage (see Authentication).

Commonly misidentified lines (See ICLAC register)	No commonly misidentified lines were used in this project.

Human research participants

Policy information about studies involving human research participants

Population characteristics	Patients included in the study were RIG patients with tumor material available for study diagnosed at Children's Hospital Colorado, St. Jude Children's Research Hospital, the Childhood Cancer Survivors' Study, and the University of Florida from 1981-2015. The population characteristics, including previous diagnoses, are identified in Table 1 and Supplemental Tables 1 and 5.
Recruitment	Patients were selected from those enrolled in tumor banking studies at each institution that are offered to all patients and in which greater than 90% enroll. We have not found any systematic bias to the population that does not enroll, making self-selection bias very unlikely to affect study results. No distinction on the basis of sex was made during recruitment; however, sex was not included in the data following deidentification to protect patient privacy.
Ethics oversight	Colorado Multi-Institutional Review Board 9COMIRB 95-500); SJCRH IRB Number: Pro----7403' Mnemonic: ZPD17-029; Reference Number: 006128. All participants were consented for tissue as a part of institutional tissue banking protocols.

Note that full information on the approval of the study protocol must also be provided in the manuscript.

Clinical data

Policy information about clinical studies

All manuscripts should comply with the ICMJEguidelines for publication of clinical research and a completedCONSORT checklist must be included with all submissions.

Clinical trial registration	Not applicable
Study protocol	Not applicable
Data collection	Not applicable
Outcomes	Not applicable
